# Supplementary material for: SLC35F2, a Transporter Sporadically Mutated in the Untranslated Region, Promotes Growth, Migration, and Invasion of Bladder Cancer Cells
Source: Cells. 2021 Jan 6;10(1):80. doi: 10.3390/cells10010080 (PMC7825079; doi:10.3390/cells10010080)
Supplement: Supplementary file 1 [file cells-10-00080-s001.zip › Supplementary-tables-and-figures_Rev2_proofread.pdf]

Supplementary Data

Table S1
Table S2
Table S3
Figure S1
Figure S2
Figure S3

Table S1. Sequences of primers for Expression analyses of target genes by RT-qPCR

Target gene	Primer	Sequence (5' – 3')
RPS13	forward	CGAAAGCATCTTGAGAGGAACA
	reverse	TCGAGCCAAACGGTGAATC
RPS23	forward	TGGAGGTGCTTCTCATGCAA
	reverse	AATGGCAGAATTTGGCTGTTTG
SLC35F2	forward	TGGCATTTCGATCAGGCAGT
	reverse	TCTGCTAGTCCCAGCAGGAT
NLucP	forward	GGGAGGTGTGTCCAGTTTGT
	reverse	CTTCAGCCCATTTTCACCGC
Luc2	forward	CGCCATTCTACCCACTCGAA
	reverse	TGTCCACCTCGATATGTGCG

Table S2. Mutagenesis primers for UTR of SLC35F2

Insert	Primer	Sequence (5' – 3')
**3'UTR-SLC35F2 mutation C/T**	forward	AGGGGCAGCTTACGGTGGCTC
	reverse	GTTCCTGACCACCAAGCCC
**3'UTR-SLC35F2 mutation C/G**	forward	ATAGCAGACTGAGAATGGACACTC
	reverse	TTCCCCAAGTGTCCCCTC

Table S1. Comparison of known frequent mutations in bladder cancer with our study

Gen	Exome seq. TCGA Cell 2017 412 MIBC	RNA seq. Hedegaard et al. Cancer Cell 2018 460 NMIBC	Exome sequencing non-silent mutations in CDS in this study		
			all cases n=20	NMIBC n=8	MIBC n=11
TP53	**48**	8	45	25	54.5
KMT2D	**28**	<5	30	37.5	27.3
KDM6A	**26**	22	30	37.5	27.3
ARID1A	**25**	13	20	12.5	27.3
PIK3CA	**22**	23	20	12.5	27.3
KMT2C	**18**	<5	10	0	18.2
RB1	**18**	7	5	12.5	0
EP300	**15**	15	10	12.5	12.5
FAT4	**15**	<5	10	12.5	12.5
FGFR3	15	**53**	5	12.5	0
KIAA1109	8	**37**	10	12.5	9.1
SYNE2	14	**31**	10	12.5	9.1
AHNAK	12	**27**	5	12.5	0
DST	9	**26**	5	0	9.1
RNF213	5	**25**	0	0	0
BIRC6	11	**25**	10	12.5	9.1
DYNC1H1	6	**25**	0	0	0
AKAP9	8	**25**	5	12.5	0

Bold: Most frequent mutated genes in MIBC (≥15%) in the TCGA Study (Comprehensive Molecular Characterization of Muscle-Invasive Bladder Cancer. Cell. 2017 Oct 19;171(3):540-556.e25. doi: 10.1016/j.cell.2017.09.007) and in NMIBC (≥25%) in the Uromol Study (Comprehensive Transcriptional Analysis of Early-Stage Urothelial Carcinoma. Cancer Cell. 2016 Jul 11;30(1):27-42. doi: 10.1016/j.ccell.2016.05.004.

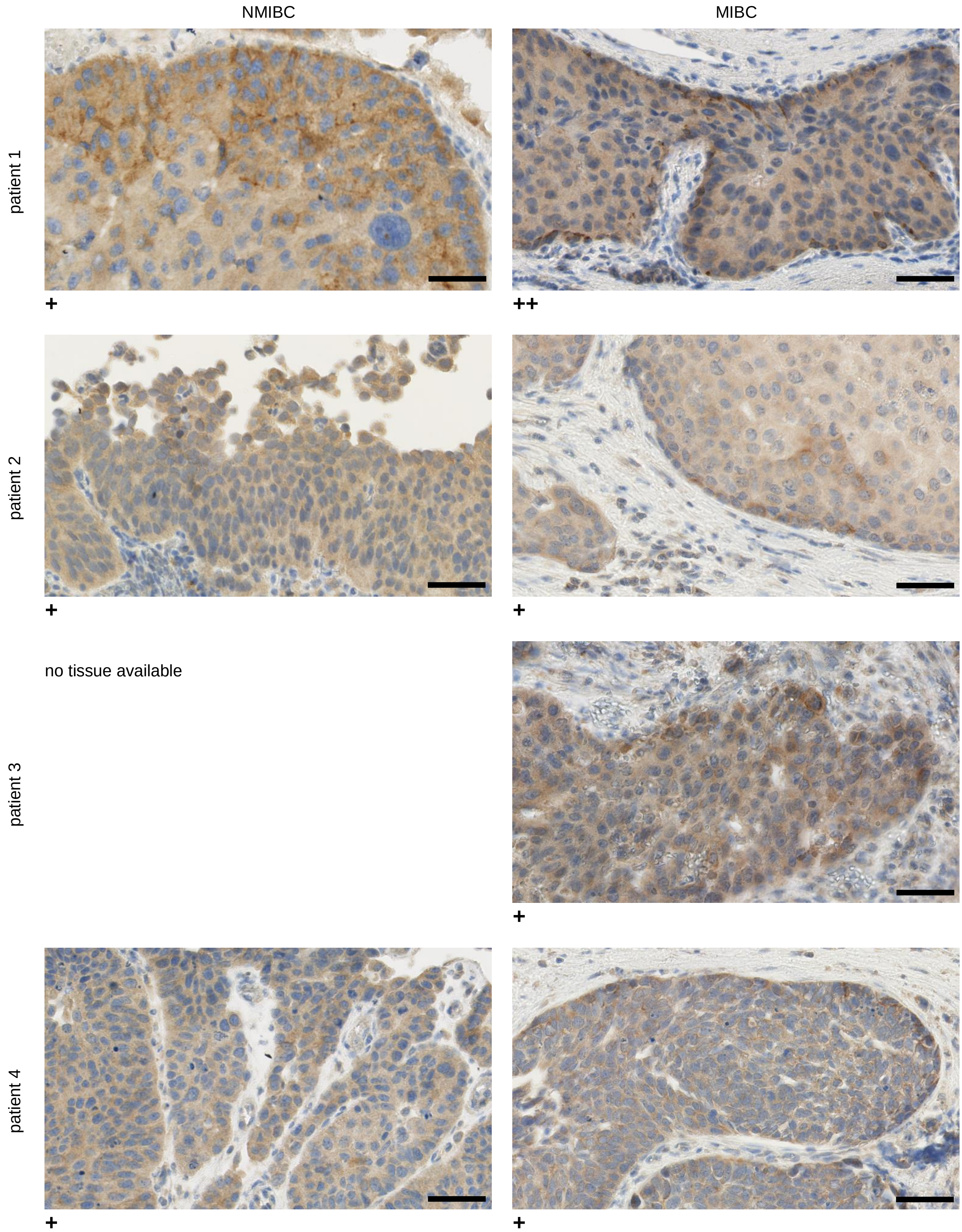

Fig. S1 Continuing on next page

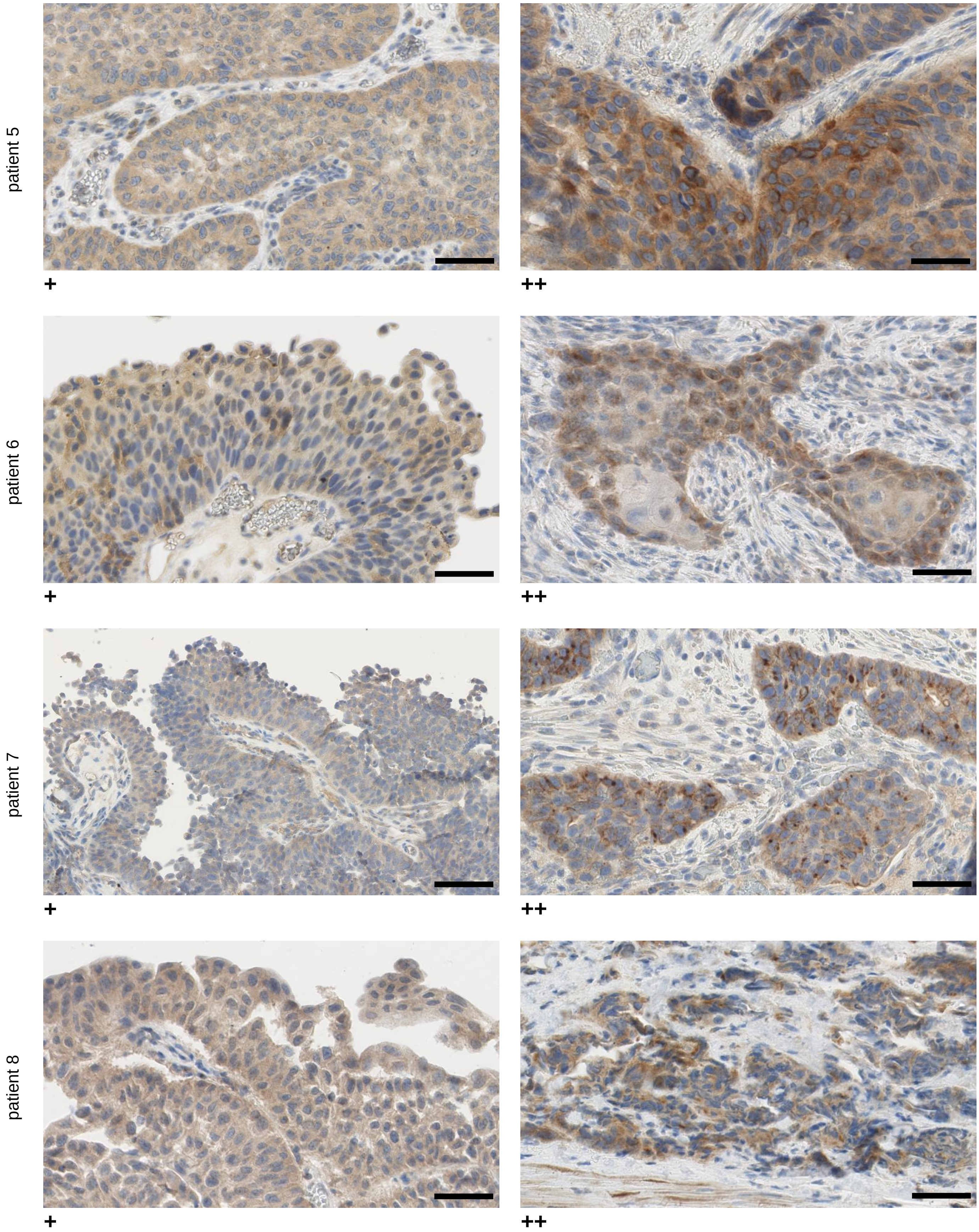

Fig. S1 Immunohistochemical staining of SLC35F2 in NMIBC and metachronous MIBC. Scale bar = 50 µm. Overall average staining intensity of the tumor cells of the whole slide is specified as negative (-), weak (+), moderate (++) and strong (+++).

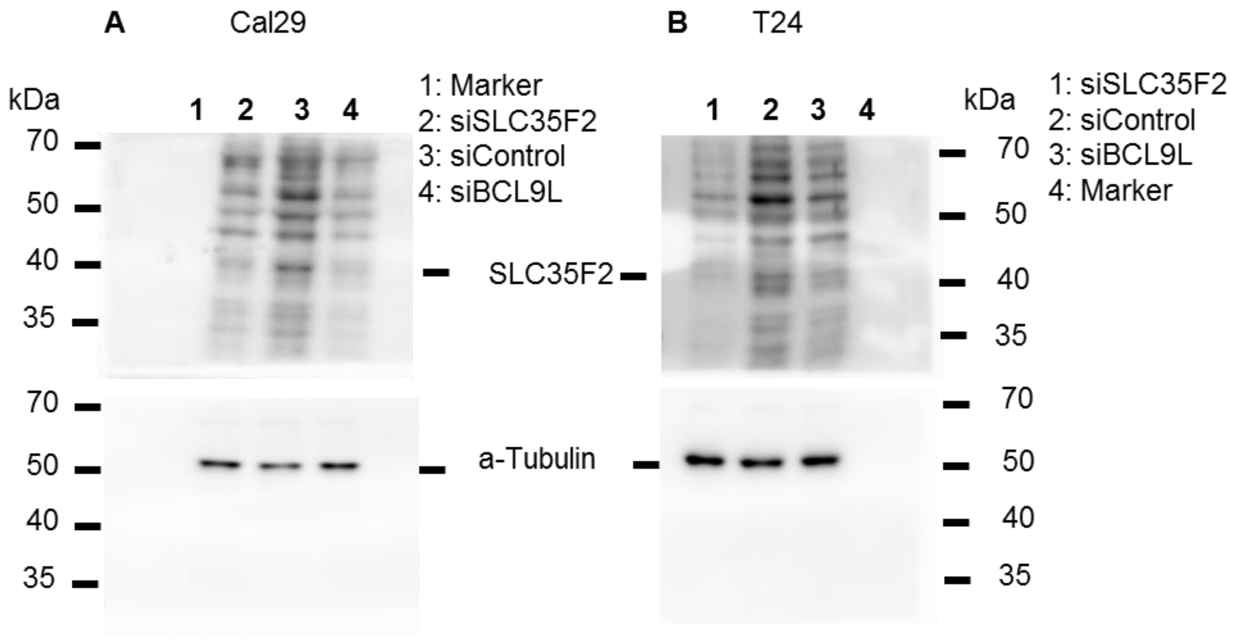

Fig. S2

Whole westernblot on PVDF membrane to estimate the protein expression of SLC35F2 after treatment of Cal29 (A) and T24 (B) cells with siSLC35F2 (Fig. A lane 2, Fig. B lane 1) or scramble siRNA (Fig. A lane 3, Fig. B lane 2) or another siRNA against BCL9L (Fig. A lane 4, Fig B lane 3). Membrane was first stained against SLC35F2 (upper) and secondly stained against a-Tubulin (lower).

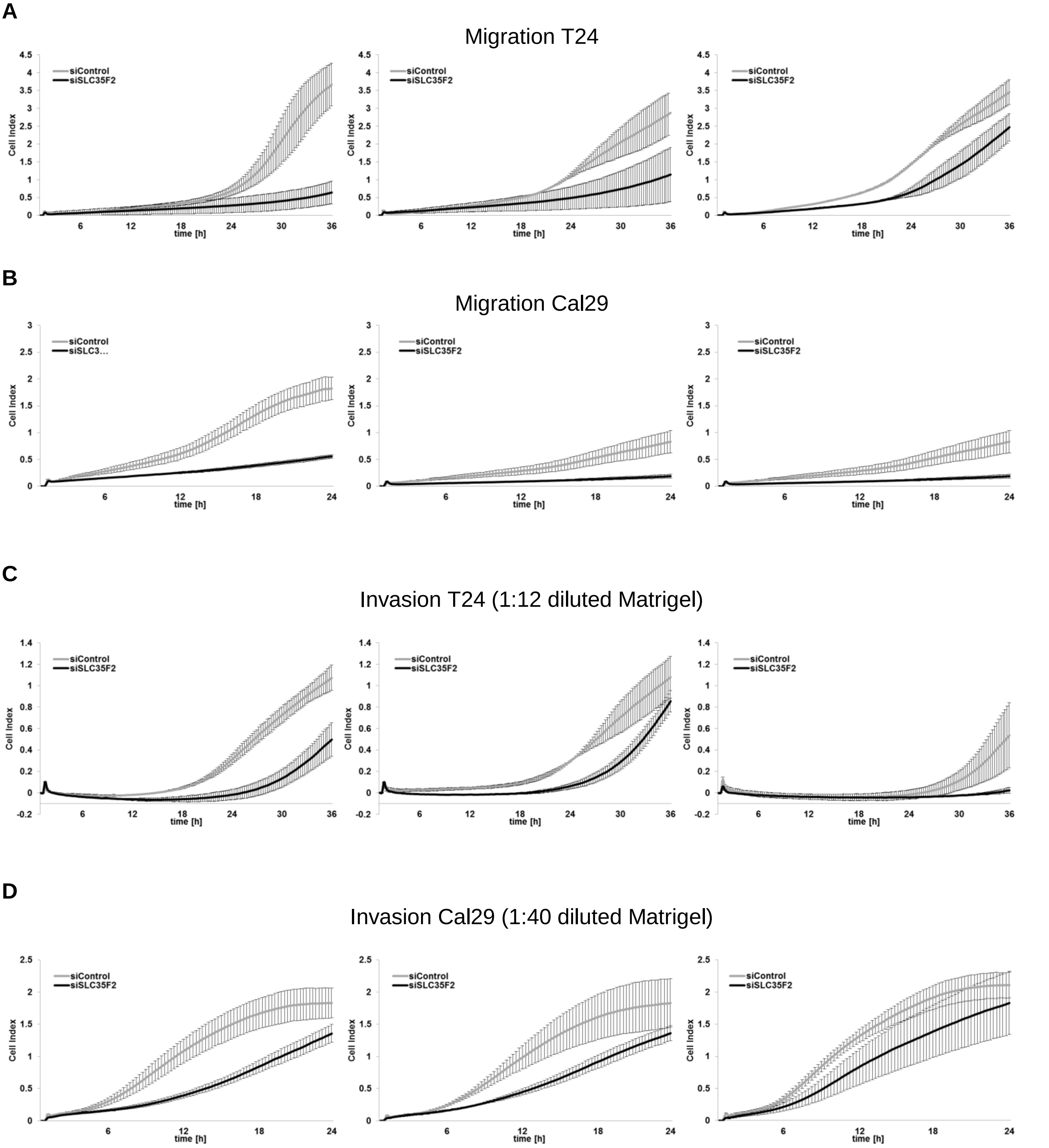

Fig. S3 SLC35F5 dependent migration and invasion of bladder cancer cells
Biological triplicates of xCelligence migration and invasion assay. Migration of Cal29 (A) and T24 (B) is reduced after siRNA knockdown of SLC35F2. Invasion was analyzed using 1:12 diluted matrigel for T24 (C) and using 1:40 diluted matrigel for Cal29. The invasiveness of T24 (C) and Cal29 (D) was significantly reduced after knockdown of SLC35F2. The cell index reflects the amount of cells that pass the membrane between upper and lower chamber.
