# Supplementary material for: SLC35F2, a Transporter Sporadically Mutated in the Untranslated Region, Promotes Growth, Migration, and Invasion of Bladder Cancer Cells
Source: Cells. 2021 Jan 6;10(1):80. doi: 10.3390/cells10010080 (PMC7825079; doi:10.3390/cells10010080)
Supplement: Supplementary file 1 [file cells-10-00080-s001.zip › Supplementary-bioinformatics-methods.pdf]

SLC35F2, a transporter sporadically mutated in the UTR, promotes growth, migration and invasion of bladder cancer cells

Supplementary bioinformatics material and methods

Whole-exome sequencing read preprocessing and mapping
Raw whole-exome sequencing reads were quality controlled with FastQC [1] (v0.11.3) and trimmed with Prinseq [2] (v0.20.3) to remove low-quality nucleotides ($Q \geq 20$, length ≥ 20). Multi-mappings of the high-quality paired-end reads were performed with Tophat [3] (v2.0.11, default parameters) against the human genome version GRCh38.80, downloaded from Ensembl. The resulting mappings were split for unique and multiple mapped reads using the ViennaNGS [4] toolbox (v0.10).

Recalibration of unique mapped reads
Prior to the call of variants in the unique mapped read data we followed the Genome Analysis Toolkit (GATK) best practice protocol [5] to realign INDELs and recalibrate base qualities with GATK (v3.4.46). First, we used the Picard [6] (v1.83) suite to reformat the alignments, followed by the INDEL realignment and base quality recalibration with GATK.

Detection of somatic variants
We used LoFreq [7] (v2.1.2) on the recalibrated alignments to call somatic variants (SNVs and INDELs) between the corresponding MNC and tumor samples for each patient, respectively. The advantage of LoFreq is the very sensitive variant-call to infer SNVs and INDELs from next-generation sequencing data. LoFreq automatically adjusts errors inherent in sequencing methods, like mapping or base/INDEL alignment uncertainties. The detected variants were further visualized and partially inspected with the help of the IGV [8] (v2.3.57) browser.

References

[1] S Andrews et al. FastQC: A quality control tool for high throughput sequence data. Reference Source, 2010.

[2] Robert Schmieder and Robert Edwards. Quality control and preprocessing of metagenomics datasets. Bioinformatics, 27(6):863{864, 2011.

[3] Cole Trapnell, Lior Pachter, and Steven L. Salzberg. Tophat: discovering splice junctions with rna-seq. Bioinformatics, 25(9):1105{1111, May 2009.

[4] Michael T. Wol_nger, J◎org Fallmann, Florian Eggenhofer, and Fabian Amman. ViennaNGS: A toolbox for building e_cient next-generation sequencing analysis pipelines. F1000Research, 2015.

[5] Geraldine A Auwera, Mauricio O Carneiro, Christopher Hartl, Ryan Poplin, Guillermo del Angel, Ami Levy-Moonshine, Tadeusz Jordan, Khalid Shakir, David Roazen, Joel Thibault, et al. From FastQ data to high-con_dence variant calls: the genome analysis toolkit best practices pipeline. Current Protocols in Bioinformatics, pages 11{10, 2013.

[6] Picard: A set of command line tools (in Java) for manipulating high-throughput sequencing (HTS) data and formats such as SAM/BAM/CRAM and VCF. http://picard.sourceforge. net. Accessed: 2016-06-06.

[7] Andreas Wilm, Pauline Poh Kim Aw, Denis Bertrand, Grace Hui Ting Yeo, Swee Hoe Ong, Chang Hua Wong, Chiea Chuen Khor, Rosemary Petric, Martin Lloyd Hibberd, and Niranjan Nagarajan. LoFreq: a sequence-quality aware, ultra-sensitive variant caller for uncovering cellpopulation heterogeneity from high-throughput sequencing datasets. Nucleic acids research, page
gks918, 2012.

[8] Helga Thorvaldsd_ottir, James T. Robinson, and Jill P. Mesirov. Integrative genomics viewer (IGV): high-performance genomics data visualization and exploration. Brief Bioinform, 14(2):178{192, Mar 2013.
